# Gallbladder Agenesis: Must General Surgeons Have It on Their Diagnostic Algorithm?

**DOI:** 10.7759/cureus.51078

**Published:** 2023-12-25

**Authors:** Atl Simon Arias Rivera, Alan Jasqui Bucay, Ivan Mandujano, Alberto Perez Cantu, David De Leon

**Affiliations:** 1 General Surgery, Hospital Angeles Lomas, Huixquilucan, MEX

**Keywords:** mri biliary tract, biliary colic pain, extrahepatic cholestasis, gallbladder ultrasound, endoscopy ercp, gallbladder agenesis

## Abstract

Gallbladder agenesis is a rare congenital malformation that can present itself with comparable symptoms as any case of cholelithiasis. We present a case of a 76-year-old male patient without any medical background of significance who presented at the ER complaining of sudden abdominal pain that started two hours prior to his arrival. Laboratory tests were ordered and an increase in total bilirubin was noted, showing a cholestatic pattern. An abdominal ultrasound was performed where the gallbladder could not be found; therefore, an abdominal CT and an MRI were ordered, which later confirmed gallbladder agenesis. Endoscopic retrograde cholangiopancreatography (ERCP) was then performed with sphincterotomy and the patient was discharged 24 hours later without any complications noted. Gallbladder agenesis is a rare but important diagnosis that general surgeons must have in their diagnostic repertoire because of its ability to mimic acute cholecystitis or cholelithiasis. The objective of this report is to summarize the principal details of this entity.

## Introduction

Gallbladder agenesis (GA) was first described in 1701. It is a very uncommon entity that mainly affects women (3:1) with an average incidence of 13 to 65 cases per 100,000 inhabitants, appearing predominantly between the second and third decades of life [1].

In approximately 50% of all patients with GA, the illness presents itself asymptomatic; however, the other 50% tend to develop similar symptoms as those with cholelithiasis. Surprisingly, it can even present itself as a case of choledocholithiasis because of the hypertonic biliary sphincter and the consecutive biliary dyskinesia [1].

GA has been associated with the additional presence of gastrointestinal, genitourinary, and muscle-skeletal malformations. Physicians must therefore have a high suspicion index of GA while making an abdomen ultrasound in patients with these types of congenital anomalies [2].

There are no developed guidelines so far that describe the handling of this disease; however, there are studies that report the alternative of using a non-surgical approach with smooth-muscles relaxants, and the management using an endoscopic retrograde cholangiopancreatography (ERCP) coupled with a sphincterotomy [3].

## Case presentation

A 76-year-old male patient without a surgical background presented at the Emergency Room (ER) at Hospital Angeles Lomas referring to a two-hour-duration sudden abdominal pain rated at 8/10 on the visual analog scale (VAS) in the right upper quadrant of the abdomen accompanied with nausea. He also mentioned having abdominal distension for the past two months with the presentation of jaundice upon arrival at the ER.

**Figure 1:**
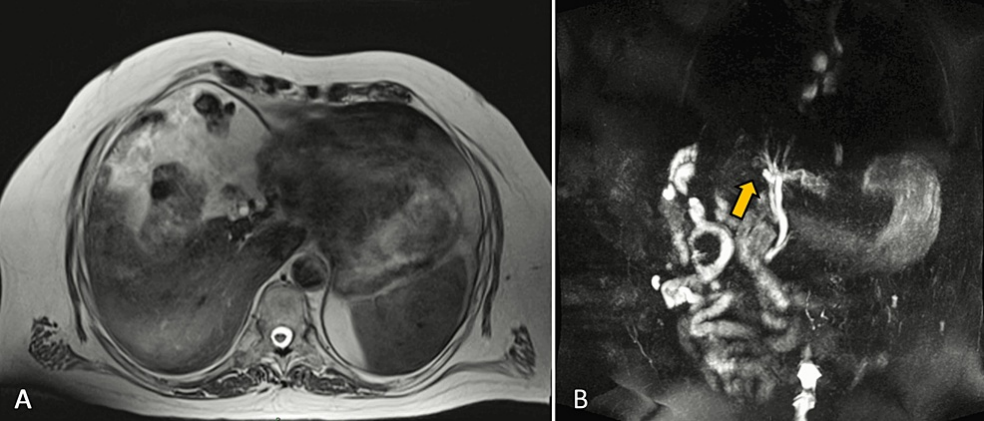
(A) MRI transversal plane with no presence of gallbladder. (B) MRI coronal plane with confirmation of gallbladder agenesis (yellow arrow).

Lab results indicated a cholestatic pattern: total bilirubin 3.0 μmol/l (direct bilirubin 2.0 μmol/l); alkaline phosphatase 350 UI/l, gamma-glutamyltransferase (GGT) 150 u/l. An abdominal ultrasound was performed, but the gallbladder could not be found by three different ultrasonologists; the common biliary duct was found intact, measuring 8 mm in diameter. An abdominal tomography was done and it reported a complete absence of the gallbladder. Finally, it was decided to perform an MRI resulting in a diagnosis of GA (Figure 1). The intrahepatic bile duct was not dilated. The common hepatic duct appeared to have a normal caliber of 0.6 cm and the cystic duct a diameter of 0.4 cm). Both of them were permeable and did not show any anomalies within their interior walls.

**Figure 2:**
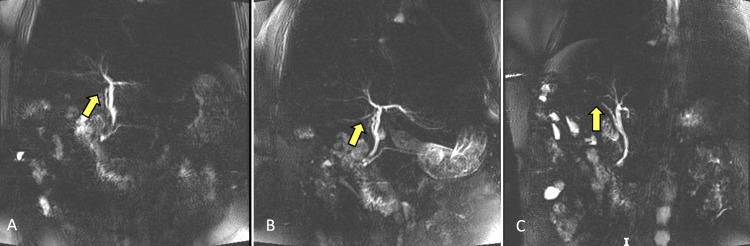
Magnetic resonance cholangiopancreatography (MRCP) showing the filling defect in the common bile duct (yellow arrow).

The bile duct had a caliber of 0.6 cm along its entire path, and in its distal and intra-duodenal portions within the cholangiographic sequence, two apparent filling defects were identified of 0.3 cm and 0.5 cm, suggesting the presence of gallstones (Figure 2).

**Figure 3:**
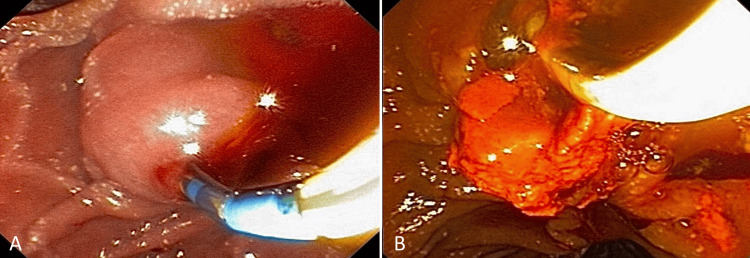
Endoscopic probe of ERCP in duodenal papilla with multiple stone extraction without any complication. ERCP: endoscopic retrograde cholangiopancreatography

To continue with the therapeutic diagnostic approach, an ERCP was performed, which then confirmed the GA diagnosis (Figure 3).

After the endoscopic procedure, the patient had an adequate evolution with resolution of abdominal pain and jaundice diminishment, with a 24-hour hospital stay, and was subsequently discharged home.

## Discussion

GA is a congenital illness caused by the lack of development or canalization of the cystic yolk during the fourth week of intrauterine life and the development of the cystic conduct during the seventh week. It is important not to think of this pathology as the inappropriate migration of the gallbladder/ectopic gallbladder, with possible locations such as: intrahepatic, underneath the postero-inferior surface of the liver, inside falciform ligament, retroperitoneal or within retropancreatic or retroduodenal areas. It presents an incidence of 10-65 cases per 100,000 habitants appearing predominantly among females with a female: male ratio of 3:1 [1-3].

This pathology was first described by Lemer in 1701 and since then it has been reported less than 500 times in global literature. It was first classified by Bennion´s classification method into three categories: (1) asymptomatic anatomical abnormalities usually diagnosed during autopsy, which constitutes 35% of cases, (2) patients who complain of pain in the upper right quadrant, dyspepsia, and jaundice, representing 50% of the cases, (3) patients who present with severe associated fetal anomalies [1-4]. In 2015, Tang et al. proposed a new classification, related to a new method that divides congenital malformations into life-threatening and non-life-threatening ones, it also corrects the overlap that exists in Bennion's classification between categories 1 and 3. So the Tang classification is: (I) symptomatic type that can be further divided into Ia and Ib subtypes where type Ia is accompanied by lethal deformities such as biliary atresia, ventricular septal defect, imperforate anus, duodenal atresia, etc. (the majority of them dying shortly after birth) and Ib, which is accompanied by non-lethal malformations such as intestinal malrotation, right liver agenesis, cryptorchism, choledocal cyst, and choledochectasia, and (II) asymptomatic type [5,6].

This pathology tends to be sporadic; however, it has been associated with other congenital malfunctions in 12-39% of the cases such as duodenal atresia, intestinal malrotation, pancreas divisum, imperforate anus, right hepatic lobe hypoplasia, duplication cysts of the hepatic angle, interventricular communication, renal agenesis, undescended testes, and syndactyly. It has also been correlated to congenital syndromes such as trisomy 18 and congenital malformations caused by in-utero exposition to thalidomide [7,8].

This pathology's diagnosis is mainly incidental during surgical procedures (50% of cases). The other remaining patients are diagnosed in the ultrasound room while taking an abdominal ultrasound for this or any other pathology suspected. The ultrasonologist will report the absence of the wall-echo-shadow-sign (the one formed by the gallbladder´s wall and the posterior ultrasonographic shadow reflected by the gallbladder's calculi) [9,10].

Even though the first ultrasound taken can be inconclusive because, in any given situation when the gallbladder is contracting or full of stones, it can be difficult to visualize it on the ultrasound, so the use of non-invasive image studies that have been proposed, such as the MRCP, is useful as a first diagnostic tool [11,12].

## Conclusions

GA is the least frequent malformation of the biliary duct. This pathology has to be taken into consideration in the scenario where the gallbladder cannot be seen through routine image methods. In the presence of this pathology, the surgeons must consider a non-surgical approach to patient management. This can avoid surgical intervention and complications that could be prevented.
